# Notes and News

**Published:** 1892-09-03

**Authors:** 


### NOTES AND NEWS.

COLLECTED AND COMMUNICATED.

A SALVE has been offered to the wounded feelings of the inhabitants of Tottenham. The fever hospital to be built there is not to be called the Tottenham, but the North-Eastern Hospital.

THE St. Anne's Convalescent Home, at Bridlington Quay, appears to be a great boon to Hull and its district, which have sent over 100 patients to the institution during the past twelve months. The home has accommodation for 135 adults and 30 children, the rooms for the latter being provided with dolls' houses and numerous toys.

STRONG feeling has been aroused over the question of holding a hospital parade in Hertfordshire. The desire to have a parade, with a view to collecting funds for the infirmary and the convalescent home, appears to be general, but opinion as to the day on which it is to be held differs considerably, and the matter is still exercising the minds of the inhabitants. What has caused so much commotion is the suggestion that the parade should be held on a Sunday, and the conflicting opinions as to the desirability of other days have led to a compromise, deferring the matter to a committee for decision. The lot of the committee does not appear to be a very happy one.

WE are daily hearing of fresh cases of cholera at some of our seaports, and in every instance the people attacked have been emigrants coming from foreign ports known to be ravaged by this disease. It is pertinent, therefore, to ask why the Government should allow vessels coming from infected ports, even with clean bills of health, to land their living cargo when the danger is so great? We have it in our power, so far as man's precautions can prevent disease, to keep this island free from the scourge, and if the disease takes firm hold of some of our towns we shall have to thank the supineness of a Government department which places the convenience of pauper and alien emigrants before the welfare of the State. Dr. Simpson, of Calcutta—an authority on this matter—has clearly pointed out the risk we are incurring by not stopping all this emigrant traffic at once.

IT appears from the report presented to the annual meeting of the Herts Convalescent Home, situated at St. Leonards, that there is scarcely a parish or district in the county which has not availed itself of the advantages of the institution. This is striking testimony to the need and usefulness of the home. The adoption of the recommendation of the Committee to reduce the weekly payment from the patients from 5s. 6d. to 4s. has been fully justified. Not only has the reduction simplified the facilities more especially for patients in securing the benefits of the institution, but the financial position of the charity has not been impaired by the step. More sitting-room accommodation has been supplied for the male patients, who have derived therefrom considerable extra comfort, and a reduction in the expenses has helped to bring the benefits of the home within the reach of nearly all the poor of the county.

IT appears that during the present month four dispensaries in Edinburgh have been closed, thereby throwing a vast amount of extra work on a fifth institution, and causing much inconvenience to the poorest classes of the community, for which these institutions are intended. No doubt there are good causes for the closing of the dispensaries, but surely it might have so been managed that each one should have shut its doors at a different time. Those who maintain, with, perhaps, some show of reason, that indiscriminate relief is the chief result of the establishment of so many institutions of this nature, will possibly see little to complain of in the closing of four out of five of them at the same time. But even if it is granted that the system of relief is abused, the question is one of management. Either the dispensaries are needed or not needed; if the former, then the greatest emergency only would justify the step taken; if the latter, the fault lies in their establishment.

THE Board of Management of the Royal Albert Edward Infirmary for Wigan and the district, is considerably exercised as to the debt on the building fund of the institution, which has been reduced by means of donations during the past few months amounting to £4,000. The debt now stands at £3,500. Towards this balance, a generous but anonymous donor has kindly promised £1,000, conditionally that the remainder is raised by November 12th next. The need for help is urgent, to enable the Board to secure the benefits of this generous donation. The necessity for funds to enable the Board to erect a suitable mortuary chapel to replace the temporary premises erected nineteen years ago, which, through lack of funds, have been allowed to remain in existence, to the discredit of the hospital, has been made known; and it is hoped that some generous benefactor will relieve the funds, by undertaking to erect a building properly adapted to the purpose. The endowment fund is but small, consequently the annual income for maintenance purposes has to be raised by extraneous means. The working classes are doing excellently; they are generously contributing one halfpenny per week, or a penny per fortnight, with the object of raising £4,500. The Secretary, Mr. W. Taberner, will most gratefully acknowledge any donations forwarded to him to help in the present emergency.

A very pretty cottage hospital, founded in memory of the late Miss Mary Hewetson, has been opened at Keswick. It is surrounded by beautiful grounds, and all that is now required to complete its comforts is some pictures on the walls and a library for the use of the patients. Accommodation is provided for ten beds, all on the ground floor, and the male and female wards are divided from one another by a central vestibule and hall. On each side of the main entrance are a large and a small ward separated by a nurse's room. In each of the large wards there are four beds, and in each of the smaller ones one. The bed-rooms for the Matron and servants are on the first floor, and the operating-room and the kitchen department are placed at the back of the building.

---

Owing to the success of the centenary bazaar held at the close of last year the Board of the Belfast Royal Hospital were enabled to present a very satisfactory report at the recent quarterly meeting. This success enabled the authorities to pay up all the accounts against the institution up to the close of July. On the other hand, the ordinary receipts fail to show any elasticity except in one direction, which is highly creditable to the working classes of Belfast. Whereas the general subscriptions show a falling off, those from the working classes show a marked increase. Belfast has the deserved reputation of being a wealthy city, but there is evidently a lack of interest, to put it mildly, amongst a section of society which, if it cannot set an example as it should, will, it is to be hoped, speedily make amends by aiding liberally a work which appeals, and should not appeal in vain, to all men of means.

---

The want of accommodation in the Isle of Wight for the isolation of cases of infectious disease, except at Ryde, is now attracting the attention of the Board of Guardians of the island. In the hospital at Ryde there is accommodation only for six male and a similar number of female patients. However healthy the island may be, it is evident that it is not equipped for emergencies, and that there is room for one if not more new hospitals. The fact that disease is frequently spread through the inability to remove a patient should not be lost sight of, but at present nothing decisive has been done. But it is satisfactory to note that increasing interest is being taken in the question, and though concensus of opinion is not yet unanimously in favour of erecting a new isolation hospital the discussion, which has recently taken place shows that it is far from averse to the proposal.

A Quarterly General Court of the Governors of the Middlesex Hospital was held on 25th inst. Mr. Henry N. Custance occupied the chair, and there was a fair attendance. The report of the proceedings of the weekly Board during the past quarter was read by the Secretary Superintendent. It stated that the new Children's Ward was now complete, and that the £1,000 promised by Mr. Pinder Simpson, the executor of the late Mrs. Holme, to defray the expense, had been duly received; several valuable legacies had also been added to the funds since the last Quarterly Court, the principal one being £7,426 Great Western Railway Five per Cent. Stock, bequeathed by the late Mrs. Isabella Chandless. The Report also called attention to the continued decrease in the receipts of the Samaritan Fund, and it was decided to devote the proceeds of the alms-boxes within the hospital to that fund in future. The Court sanctioned the expenditure of various sums on certain much-needed improvements and repairs, among them being a set of additional baths for the patients.

